# From nuclear track characterization to machine learning based image classification in neutron autoradiography for boron neutron capture therapy

**DOI:** 10.1371/journal.pone.0293891

**Published:** 2023-12-21

**Authors:** Julia S. Viglietti, María S. Espain, Rodrigo F. Díaz, Luis A. Nieto, Manuel Szewc, Guillermo C. Bernardi, Luis M. Rodríguez, Daniel E. Fregenal, Gisela Saint Martin, Agustina M. Portu

**Affiliations:** 1 Departamento de Radiobiología, Centro Atómico Constituyentes, Comisión Nacional de Energía Atómica (CNEA), Buenos Aires, Argentina; 2 Consejo Nacional de Investigaciones Científicas y Técnicas (CONICET), Buenos Aires, Argentina; 3 Escuela de Ciencia y Tecnología (ECyT), Universidad Nacional de San Martín (UNSAM), Buenos Aires, Argentina; 4 International Center for Advanced Studies (ICAS) and ICIFI (CONICET), ECyT-UNSAM, Campus Miguelete, Buenos Aires, Argentina; 5 Gerencia de Tecnología de la información y de las Comunicaciones (GTIC), Subgerencia Vinculación y Desarrollo de Nuevas Tecnologías de la Información, DCAP-CNEA, Centro Atómico Constituyentes, Buenos Aires, Argentina; 6 Department of Physics, University of Cincinnati, Cincinnati, Ohio, United States of America; 7 Centro Atómico Bariloche, Comisión Nacional de Energía Atómica (CNEA), Bariloche, Argentina; Universiti Teknologi Malaysia, MALAYSIA

## Abstract

Knowledge of the ^10^B microdistribution is of great relevance in BNCT studies. Since ^10^B concentration assesment through neutron autoradiography depends on the correct quantification of tracks in a nuclear track detector, image acquisition and processing conditions should be controlled and verified, in order to obtain accurate results to be applied in the frame of BNCT. With this aim, an image verification process was proposed, based on parameters extracted from the quantified nuclear tracks. Track characterization was performed by selecting a set of morphological and pixel-intensity uniformity parameters from the quantified objects (area, diameter, roundness, aspect ratio, heterogeneity and clumpiness). Their distributions were studied, leading to the observation of varying behaviours in images generated by different samples and acquisition conditions. The distributions corresponding to samples coming from the BNC reaction showed similar attributes in each analyzed parameter, proving to be robust to the experimental process, but sensitive to light and focus conditions. Considering those observations, a manual feature extraction was performed as a pre-processing step. A Support Vector Machine (SVM) and a fully dense Neural Network (NN) were optimized, trained, and tested. The final performance metrics were similar for both models: 93%-93% for the SVM, vs 94%-95% for the NN in accuracy and precision respectively. Based on the distribution of the predicted class probabilities, the latter had a better capacity to reject inadequate images, so the NN was selected to perform the image verification step prior to quantification. The trained NN was able to correctly classify the images regardless of their track density. The exhaustive characterization of the nuclear tracks provided new knowledge related to the autoradiographic images generation. The inclusion of machine learning in the analysis workflow proves to optimize the boron determination process and paves the way for further applications in the field of boron imaging.

## Introduction

Solid State Nuclear Track Detectors (NTDs) have been widely used to detect ionizing radiation in multiple application fields, such as nuclear physics, dosimetry, astrophysics, radon detection, radiopharmaceutics, and Boron Neutron Capture Therapy (BNCT) [1–4]. These detectors are in general good thermal and electrical insulators, and have the ability to register damage produced by heavy charged particles in a permanent way. Incident ions deposit their energy along the trajectory inside the detector, causing excitations and ionizations. For polymeric detectors this process leads to broken polymeric chains and reduced molecular weight in the damaged region [5]. Damage paths, with diameters of the order of nanometers, also called latent tracks or pits, can be amplified by treating the detector with a suitable chemical solution. The solution etches out the material from the damaged trail at a faster rate (with a velocity V_T_) compared to the surrounding undamaged material (with velocity V_B_). Track pit shape is mainly determined by the ratio (V) between V_T_ and V_B_, so when V>1, tracks are enlarged and, depending on the etching time, can be observed under optical microscopy.

In particular, when a sample containing a charged-particle emitter is put in contact with a NTD, the assessment of etch-pits sites provides information about the spatial distribution of the element in the sample. This application is of particular interest for BNCT, a selective and binary modality of radiotherapy that has been successfully applied to multiple tumour targets [6]. The therapy is based on the administration of a ^10^B enriched compound, which is absorbed preferably by neoplastic cells, and the subsequent irradiation with thermal-epithermal neutrons. Due to the behaviour $\approx 1/\sqrt{E}$ of the ^10^B(n,*α*)^7^Li (BNC) reation’s cross section for low energy neutrons, the BNC reaction takes place (Table 1). Consequently, the ^4^He and ^7^Li ions are ejected in practically opposite directions, depositing their energy in a short range (about the diameter of one cell). The effectiveness of BNCT depends on the selective and homogeneous accumulation of ^10^B atoms within the cancer cells. Hence, the knowledge of ^10^B concentration and microdistribution in tumour and surrounding tissue is of great relevance not only when planning a treatment, but also for evaluating the biological effectiveness of different boron compounds.

**Table 1:** Decay channels for the ^10^B(n,*α*)^7^Li reaction.

Particle	Energy	Intensity
*α*_0_	1.777 MeV	6.3%
^7^Li_0_	1.013 MeV	
*α*_1_	1.471 MeV	93.7%
^7^Li_1_	0.839 MeV	

Within this context, neutron autoradiography on NTDs is widely applied to study boron microdistribution in tissue samples previously infused with a boron carrier [7–9]. In our group, different approaches to neutron autoradiography have been developed and applied in various biological models using polycarbonate as NTD [10–13]. By irradiating a boronated sample adhered to an NTD with a neutron flux, *α* and ^7^Li particles penetrate the detector and create latent tracks. After the chemical attack, tracks become observable under optical microscopy. Since ejected particles have a short range from the reaction site, pits in the acquired autoradiographic images are associated with boron distribution in the samples. The correlation between track density in different tissue structures and boron concentration is possible due to calibration curves generated from boronated standards of known concentration [14].

Since ^10^B concentration depends on the correct track density quantification, image acquisition and processing conditions should be controlled and verified, in order to obtain accuracy and repeatability. This is not a trivial task, since images are a result of the optical effect of the damages (holes) left by charged particles, but also by any indentation, mark, or fold produced during the experimental set-up. The main criterium for distinguishing etch-pits from artifacts is that the etch-pits have regular geometrical shapes (circular or elliptical depending upon the angle of incidence) and rather uniform grayscale values. The study of the observable characteristics of the nuclear tracks is of interest for a further understanding of the experimental process behind their formation. In fact, the use of morphological parameters for the identification and quantification of BNC events has been reported [9, 15, 16]. Considering these aspects, the number of valid events (absence of artifacts) is expected to be greater in images acquired under adequate conditions as opposed to those obtained, for example, under poor illumination. However, the experimental process occasionally makes it difficult to obtain perfect images. For this reason, image classification using feature extraction methods was a promising idea for rejecting inadequate images.

Over the last few years, machine learning-based methods have become widely popular in the fields of life and medical science [17–19]. Their application for medical imaging in clinical and research settings are transforming the way the structural and metabolic information is addressed [20]. At research level, both *in-vivo* and *ex-vivo* techniques take advantage of the capacity not only to classify but also to perform segmentation and tracking objects [21]. In the field of track identification in NTDs, different approaches have been reported recently using machine learning algorithms [22–25].

During the training step, classification algorithms such as Support Vector Machines (SVM) or Artificial Neural Networks (ANN) evaluate the model by comparing the output to the ground truths and work iteratively trying to optimize its performance. Briefly, SVMs are algorithms designed to find a hyperplane in the n dimensional feature space that maximizes the perpendicular distance between the decision boundary and the closest data point, or margin between the classes, to minimize generalization error [26]. These types of algorithms are simple and well known, so SVMs are usually selected as baseline models to compare the final algorithm’s performance. As for ANNs, they are composed of nodes or neurons connected by weight factors. This arrangement makes it possible for every neuron to have a different importance for each node in the following layer, allowing for more flexible models [27].

The work presented in this paper will be divided into two main topics. A characterization of detected events in autoradiographic images is introduced, corresponding to the distributions of relevant morphological and uniformity metrics. The purpose was to gain information on the experimental process, mediated by the appearance of the resulting tracks. For this analysis, images from multiple samples and acquired in a variety of conditions were compared. The second part deals with a classification algorithm developed to identify adequate images. By applying the results gained from the first analysis, the method employs supervised learning techniques to filter autoradiographic images. The main goal is to improve quantification of boron in tissue samples, by avoiding quantification errors due to wrongly acquired images, but without rejecting too many acceptable images.

## Materials and methods

### Sample preparation and observation

Autoradiographic images analysed in our laboratory are generated from boronated samples in contact with polymeric NTD (polycarbonate Lexan^™^ foils, 250 *μ*m thickness), which are then irradiated with a thermal neutron flux. This group will be referred to as BNC images, and those included in the present work have been obtained from different boronated matrices:

- Aqueous solutions prepared with ^10^B enriched standards, ranging from 0 to 100 *μ*g.kg^-1^ [14, 28].
- Tissue sections corresponding to biodistribution studies for BNCT [11–13]. All images were obtained from previous studies, so no laboratory animals were used for this work.
- A boronated Standard Reference Material (SRM): ^10^B implanted in a Silicon Standard surface, with a maximum concentration at 0.188 *μ*m depth. Maximum depth < 0.4 *μ*m. When the BNC reaction takes place in the material after irradiating it with thermal neutrons, this depth distribution of boron atoms leads to emitted *α* particles with an energy spectrum of approximately (1.32±0.08) MeV (2*σ*, 94%) and (1.64±0.08) MeV (2*σ*, 6%); and Li ions with an energy spectrum of approximately (0.61±0.08) MeV (2*σ*, 94%) and (0.77±0.09) MeV (2*σ*, 6%) [29].

All the BNC images came from samples processed under the same conditions, optimized for quantitative analysis in our laboratory: Lexan-sample arranges were irradiated with a thermal neutron fluence Φ_th_ = 10^12^ cm^-2^ (RA-3 nuclear research reactor at Centro Atómico Ezeiza). For biological samples, tissue sections were removed using trypsin. Finally, detectors were chemically treated with a PEW solution (90 g distilled water, 80 g ethanol y 30 g KOH) at 70°C for 2 min [14].

To further analyse the dispersion in track-parameters distributions, BNC images were compared to those generated from monoenergetic particles. Lexan foils were irradiated with monoenergetic *α* particles with nominal energies of 0.5 MeV, 1 MeV and 1.47 MeV and Φ = 10^6^ cm^-2^, at the 1.7 MV Tandem Accelerator of Centro Atómico Bariloche. All samples were processed under the same conditions as biological BNC images.

Autoradiographic images were acquired with a Carl Zeiss MPM 800 microscope, in bright field modality. This microscope is equipped with magnification lenses of 2.5x, 20x, 40x and 100x; an AxioCam MRc5 digital camera connected to a PC with an acquisition program (AxioVision Rel. 4.8) and a motorized stage with 1 *μ*m resolution. The system allows the mapping of the entire sample by setting a coordinate system, to then return to the regions of interest (ROIs) when inspecting the tracks. Track images are typically acquired under a 40x magnification in saturation conditions pre-defined as optimal for quantification (contrast = 13, bright = -90, gamma = 1). Light and focus are left as free parameters in order to compensate for irregularities on the NTD and obtain adequate images, since suitable acquisition conditions may vary in consecutive fields. After adjusting light intensity and setting the focus on the plane of the tracks, the observed area is saved as a greyscale image under a resolution of 1280x962 pixels (211x158 *μ*m). An image analysis software, Image Pro^™^, is used for object segmentation and quantification by performing a threshold-based binarization, followed by the segmentation of 8-point connected pixels [30].

**Fig 1:**
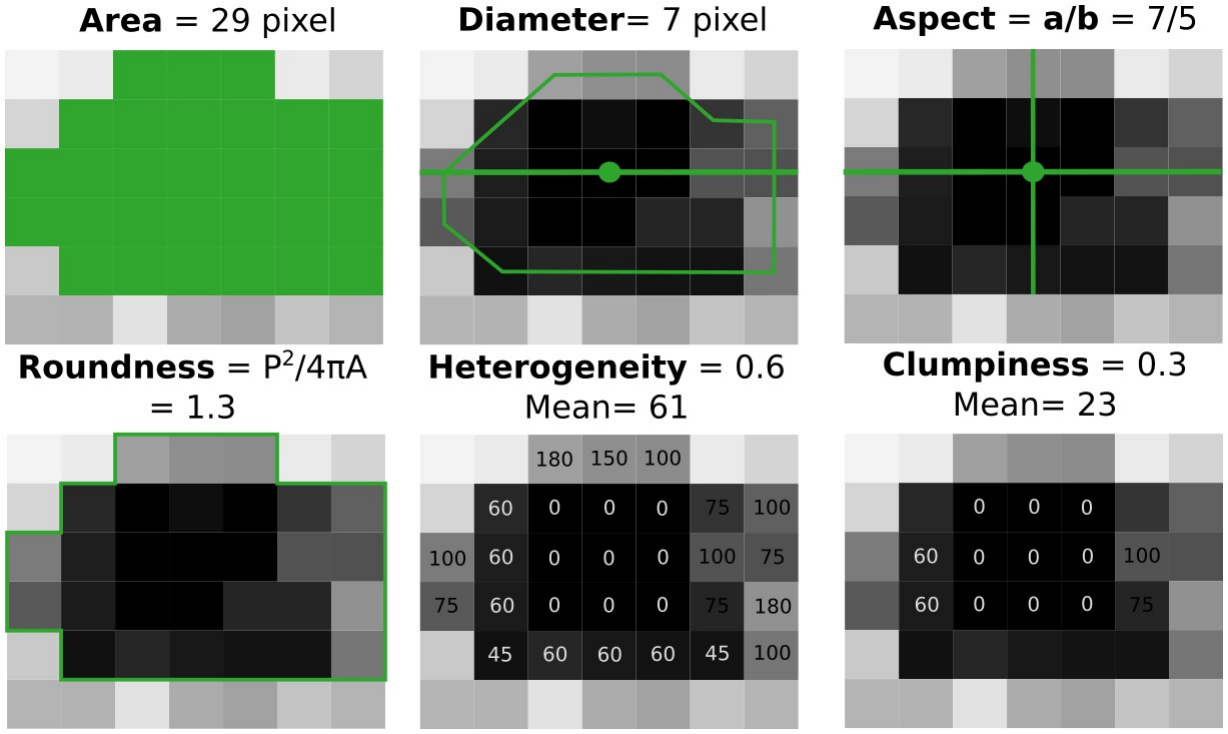
Schematic representation of the selected parameters used to represent objects detected by the ImagePro segmentation. a and b represent major and minor axes respectively, P is the object’s perimeter and A its area.

In order to characterise the autoradiographic images, a series of parameters were computed for each isolated event, chosen according to to their potential relevance for track characterization and detection of variations in the image acquisition conditions. The selected parameters (Fig 1) were:

- **Area**: Number of pixels identified as the quantified object (without considering holes within the object).
- **Diameter**: Longest diameter of the object, computed as the longest line that cuts through the centroid and connects 2 opposite border pixels.
- **Aspect ratio**: Ratio of long to short axis of the object, considering an equivalent ellipse (this way values of “1” relate to circular objects).
- **Roundness**: Measures an object’s circularity, computed as P^2^/(4*π*A), where P = perimeter and A = area (both in pixels). For a circle, P = 2*π*r and A = *π*r^2^, then, Roundness is equal to 1.
- **Heterogeneity**: Fraction of pixels with intensity values that differ by more than 10% from the mean value of the object. A value of “0” would indicate the object is homogeneous in intensity (all pixels are similar), whereas a value of “1” would indicate an extremely heterogeneous object (none of the pixels are close to the mean).
- **Clumpiness**: Reflects texture variations, defined as the fraction of pixels with intensity values differing from the mean value of the object, after performing an erosion. This parameter computes the Heterogeneity after rejecting border pixels from the object; since an intensity gradient is expected to be seen in the outer pixels of the tracks, and this parameter “erases” those border pixels, Clumpiness should present more homogeneous objects.

### Influence of light and focus settings

**Fig 2:**
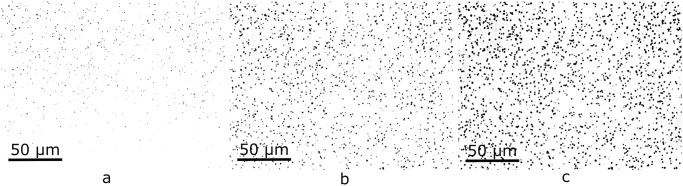
Three steps of a light intensity variation. a) exhibits an excess of illumination, b) is the representative typical image, and c) light is insufficient.

**Fig 3:**
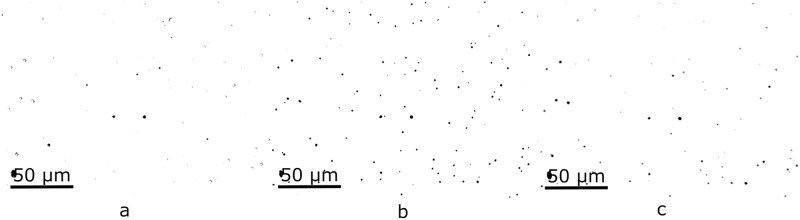
Three steps of a sample-lens distance variation. a) distance is closer than optimal, b) is the representative typical image, and c) the lens is too far from the sample to focus correctly.

For a better understanding of how acquisition conditions affect the parameter distributions, a set of images from the same area was obtained, differing from each other only by the field illumination in the microscope (Fig 2). For this purpose, conditions were set to obtain a typical image (considered “adequate”). Then, light intensity was slightly increased and decreased at regular steps, and subsequent images were acquired. This process continued until tracks could not be seen on the image. Similarly, another group of images was acquired varying the distance between the lens and the sample (Fig 3). The approach was analogous to the previous one: the light intensity was fixed for an adequate image and the distance to the sample was slightly changed between pictures.

### Dataset exploration

More than 22000 historical BNC images obtained as described in Section Sample preparation and observation were used, corresponding to aqueous solutions and biological tissues with different ^10^B concentrations. These images have been acquired by a variety of researchers over several years, resulting in a diverse and representative data set. In order to use a supervised learning approach, a label “Accepted” or “Rejected” was defined for each image, based on the classification criterion of autoradiographic images experts. A label of “Rejected” corresponds to inadequate conditions that could lead to poor and unreliable quantification results. On the other hand, the “Accepted” label corresponds to images that should be used to obtain valid results.

A set of statistical parameters (mean, median, interquartile distance, standard deviation, 5th percentile and 90th percentile) was computed over all events / tracks for all of the 6 parameters extracted from the quantified objects in each image (S1 Dataset). This led to the use of 36 features plus a label to represent each image. Since the number of images corresponding to the established classes was not balanced (53% being classified as accepted images), the dataset was reduced to 20714 examples with an equal amount of images in each class (out of which 20% was randomly selected to serve as a test set).

**Fig 4:**
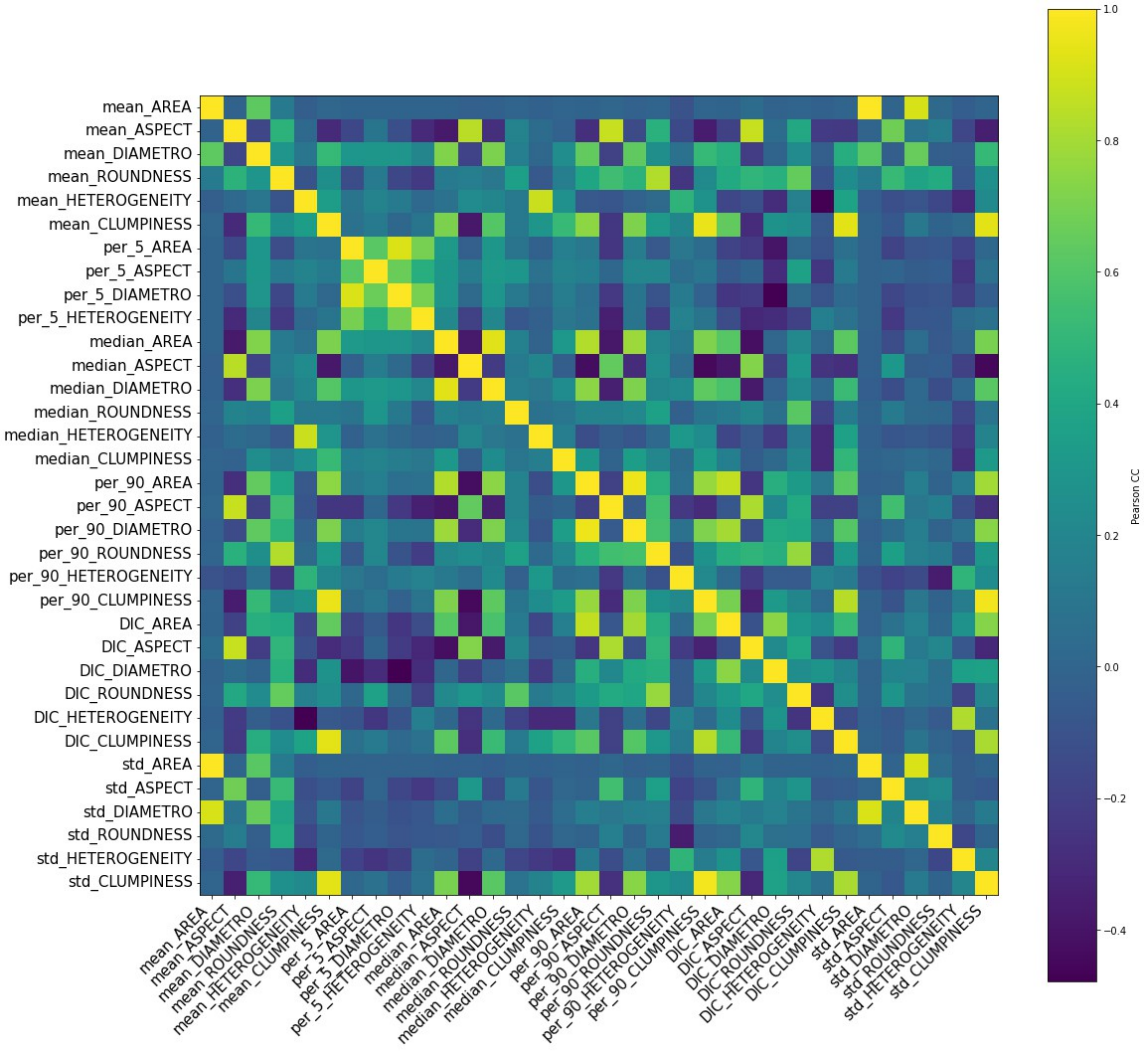
Correlation matrix for the 34 dimensions feature-space. Blue pixels represent correlation values closer to 0 (no linear correlation), while yellow pixels indicate values closer to 1.

The first step towards this machine learning approach was to evaluate the quality of information extracted from the images and their parameter distributions. For this, Pearson’s correlation coefficients were used to compute the matrix in Fig 4, where there appears to be a relevant linear correlation among many pairs of features (seen as the yellow-ish pixels outside the diagonal). Out of the 36 initial features, 2 of them (5th percentile for Clumpiness and Roundness) were initially disregarded for not contributing with useful information, since more than 5% of the quantified objects belong to the first bin in both parameters, for every analysed image in both classes.

**Fig 5:**
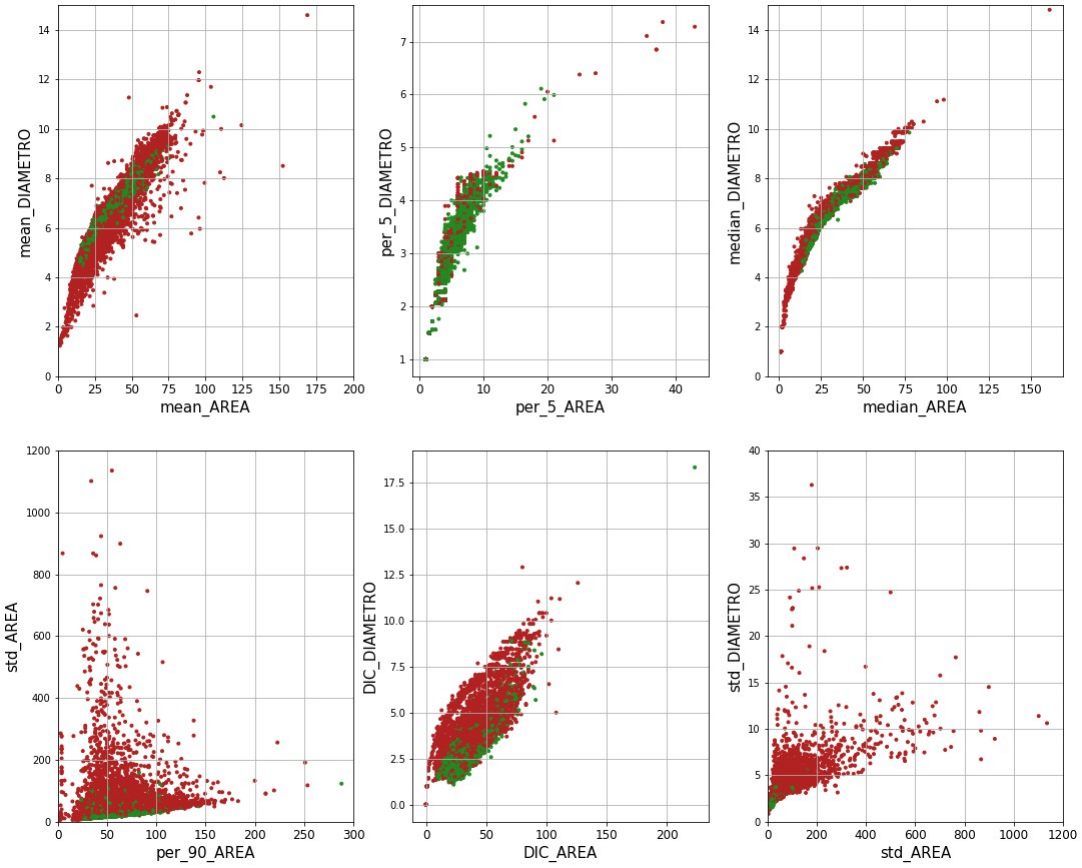
Relation between area and diameter statistical parameters. Green dots represent data points labelled as Accepted, while red ones represent Rejected labels.

Furthermore, features were compared against each other to zoom into the correlation matrix. Since the statistical parameters in Area vs Diameter were expected to show a strong correlation, they were analysed and Fig 5 confirmes this foreseen behaviour for median, mean and 5th percentile. In some cases, rejected images have a bigger deviation from linearity than accepted ones.

**Fig 6:**
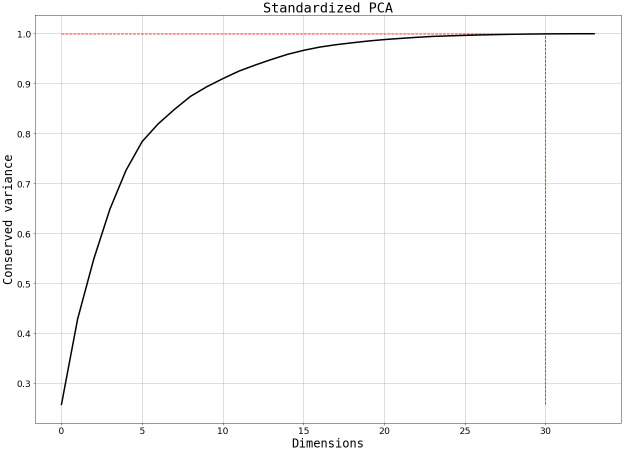
Relevant and different information (variance) conserved by adding extra PCs to the dataset. The 0.999 fraction is marked in the red dashed line.

Since correlation among features implies a level of repetitiveness of information that could be potentially counterproductive in the training step, we performed a Principal Component Analysis (PCA) to transform the feature-space in order to optimize the use of the available information. This technique finds a change of variables that makes the feature correlation matrix diagonal. In other words, it finds the eigenvector of the correlation matrix. Sorting the eigenvectors by their corresponding eigenvalue, one can choose a reduced number of vectors that keep a fraction of the data variance. This analysis can simplify the problem: by getting rid of correlated features and concentrating the feature space, the model is potentially more capable to generalize. After PCA was applied to the training set, 31 PCs were kept to maintain 99.9% of the variance (Fig 6).

### Classification algorithms

Two types of models were developed and compared, a Support Vector Machine (SVM) as a baseline model for comparison purposes, and an Artificial Neural Network (ANN). The SVM was selected as the base model out of different trained algorithms due to its simplicity.

The SVM algorithm was trained with an RBF kernel and using randomized cross-validation for finding the best hyperparameters. This approach allows the testing of multiple combinations of hyperparameters by training the model (with cross-validation) using a random selection within the pre-defined hyperparameter ranges. The best values are selected by comparing the performance of the different models. Particularly, out of N possible choices on the [20, 30], [0,0.3] ranges, the best values of C and *γ* were 16.5 and 0.09 respectively, but other combinations resulted in similar performance metrics.

For the neural network, the final architecture was defined by comparing the generalization performance, which was estimated using a validation set of 20% (out of the 80% remaining after splitting into train-test sets). The final model (Table 2) consisted of a 3 layer fully-connected network, with 150 neurons per hidden layer. A backpropagation training algorithm was selected, due to its simplicity, easy implementation and understandability. It is worth mentioning that lately, other training algorithms have emerged and are being implemented as alternatives to backpropagation, particularly in deeper and more complex networks [31].

**Table 2:** Summary of the final NN’s characteristics.

# Hidden layers (HL)	3
# Neurons per HL	150
Regularization	L2
Dropout probability	0.24 (after HLs 1 & 2)
Activation Function	ReLU + Sigmoid (output)
Learning rate	1.5e-3 (initial)
Learning rate reduction	Factor: 0.18, min change: 0.01
Optimization algorithm	Adam
Loss function	Sparse categorical cross-entropy

The training process was performed with a maximum of 1000 epochs, and a batch size of 16. To avoid overfitting, L2 regularization and dropout were used. Additionally, some callbacks were set to reduce the learning rate or stop the training process in case the loss in the validation set started to increase (after 12 or 24 consecutive epochs respectively). The training process stopped at 80 epochs.

Both algorithms were implemented using Python libraries (TensorFlow-Keras [32, 33] & Scikit-learn [34]) in a Google Colab environment. The best hyperparameters for the SVM were found using Randomized Search, whereas for the NN, they were fine-tuned by evaluating the performance on both training set and validation set. The confusion matrix and Receiver Operating Characteristic (ROC) curve were computed in the (cross-)validation set.

## Results and discussion

Parameter distributions were observed and compared for: various ^10^B concentrations, different matrices (aqueous vs. tissue), and variable acquisition conditions (adequate and inadequate). In order to complete the analysis, parameter distributions of BNC images were compared to those belonging to NTDs irradiated with monoenergetic *α* particle rays, and processed as the rest of the BNC samples.

### Characteristic parameter distributions

**Fig 7:**
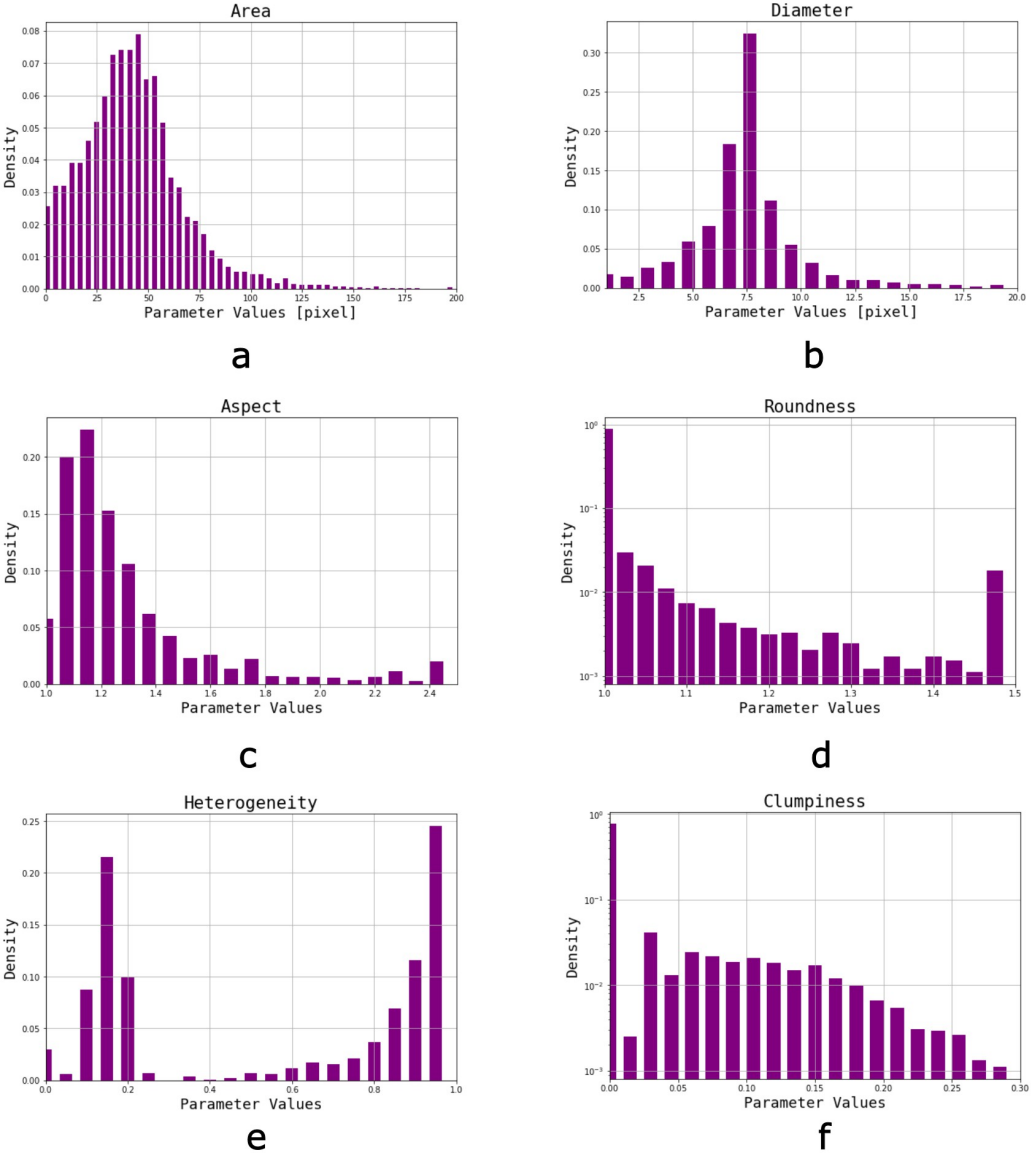
Parameter distributions of typical adequate images (14, corresponding to ≈9800 tracks) from a 60 ppm ^10^B aqueous solution. a) Area, b) Diamenter, c) Aspect ratio, d) Roundness (presented in logarithmic scale), e) Heterogeneity, f) Clumpiness (presented in logarithmic scale).

The first step for track characterization was the observation of adequate images. Multiple distributions of different sets of images were analyzed. Normalized distributions resulting from all detected objects (≈9800) in a group of 14 typical autoradiographic images belonging to an aqueous 60 ppm ^10^B solution are presented in Fig 7. Area distribution (Fig 7a) is centred around 30–40 pixels (0.86–1.15 *μ*m^2^), and shows a more marked drop towards larger sizes than to smaller ones. The first few bins resulting from images acquired in inadequate conditions usually represent background pixels or artifacts, but there is always a contribution of smaller or deeper tracks as seen in these adequate images. On the other hand, bins related to larger area values generally accumulate objects such as overlapping tracks, damages to the NTD and other artifacts generated from shadows. Diameter distribution (Fig 7b) presents a similar trend, with a predominance of tracks around 7–8 pixels (corresponding to 1.2–1.3 *μ*m).

Aspect distribution (Fig 7c) presents a peak around 1.1, plummeting for higher values which correspond to some overlapping tracks or non-perpendicular incidence angles, while there are few objects with ratios of 1. Roundness (Fig 7d) is characterized by an accumulation of objects in the first bin (1), meaning tracks are mostly circular, but there appears to be a slight accumulation of artifacts in the final bin (values>1.4), corresponding to spots or overlapping tracks. It should be noted that both Aspect and Roundness are interpretable as circularity measurements but refer to different quantities extracted from the objects, so both parameters are complementary.

Heterogeneity (Fig 7e) behaves differently than expected. In spite of seemingly uniform tracks (which would have resulted in a highest accumulation for values of 0), the distributions present two concentration areas around 0.1–0.2 and 0.9–1, both cases corresponding to objects considered as nuclear tracks but with a visible difference in pixel intensity over the edge. Clumpiness (Fig 7f) accumulates preferentially in the first bin (0), indicating that pixels within objects are similar in intensities (excluding border pixels). Both Roundness and Clumpiness distributions are presented in logarithmic scale for better visualization.

Distributions from images belonging to biological samples showed similar behaviours to the ones from aqueous solutions with comparable track density. This indicates that observations obtained from BNC images in aqueous matrices can be extended to boronated biological samples as well. On the contrary, an observable difference between high and low concentrations arose from the last bins in Area and Roundness: in images with high track density a greater amount of larger and irregular tracks can be found. Since this phenomenon is linked to overlapping tracks, a watershed split method was employed during the process of quantification to correct, to some extent, the final value. Similar methods have been employed to separate events. Eroding the image, for example, allows a better detection of central pixels [35].

### Dispersion inherent to the BNC reaction

Since BNC images are generated by *α* particles and ^7^Li ions emerging within a range of distances from the surface of the NTD, a natural variation of incident energies is expected to occur (from 1.471 MeV and 0.839 MeV respectively to virtually zero), and this results in a significant dispersion in the studied parameter values. In NTD foils irradiated with monoenergetic *α* particles deviations from the nominal energy were not expected to affect the final image. On the other hand, in the SRM most particles (both *α* and ^7^Li) originate at a fixed distance from the surface, so the travelled distance of the particles varies due to their emission angle. These distributions were compared to those produced by a typical BNC sample, an aqueous solution of 30 ppm ^10^B.

**Fig 8:**
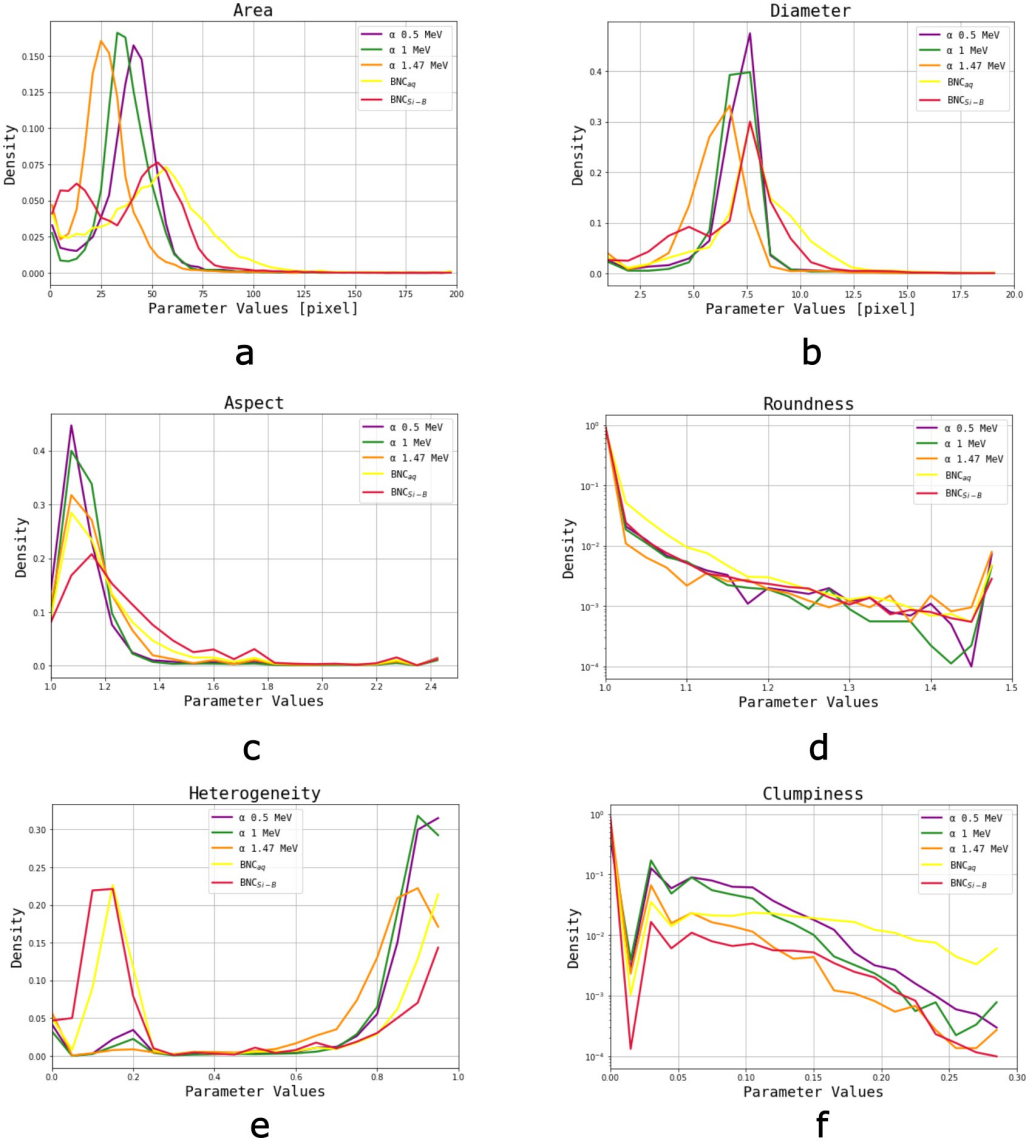
Parameter distributions of *α* particle tracks (3 nominal energies) compared to a boronated sample (SRM) and a 30 ppm ^10^B aqueous solution (similar track density). Each distribution corresponds to 45–50 images (≈200 tracks per image). a) Area, b) Diamenter, c) Aspect ratio, d) Roundness (presented in logarithmic scale), e) Heterogeneity, f) Clumpiness (presented in logarithmic scale).

Area distributions (Fig 8a) belonging to monoenergetic *α* particles have a smaller dispersion than BNC’s, and the different energies can be set apart. There appears to be a shift towards smaller values for higher energies (means at 25<33<41 pixels for 1.47>1>0.5 MeV respectively), which could be explained by the energy deposition mechanism in the NTD. Heavy charged particles traversing matter lose energy primarily through the ionization and excitation of atoms. The stopping power is defined as the mean energy loss per unit path length (–dE/dx) and it describes the way heavy charged particles deposit their kinetic energy in the surrounding medium. It increases gradually while charged particles lose energy, and for this reason most of their energy is deposited near their range (Bragg peak). Therefore, less energetic *α* particles may produce a greater amount of ionizations and excitations that result in a bigger damage zone, than the more energetic ones.

On the other hand, the area distribution of the boronated Si foil presents two peaks, one for *α* particles and one for Li ions, corresponding to sizes of approximately 12 pixels and 55 pixels respectively. Even though most particles are emitted at the same distance from the detector, the angle with which they emerge determines the length of the path they travel, and consequently their residual energy when they reach the NTD. Thus, there is an observable widening in area distribution compared to monoenergetic irradiations, but this dispersion is not enough so as to completely overlap the two particle distributions. These observations cannot be so clearly applied to Diameter distributions (Fig 8b).

Aspect peak corresponding to 1 and 0.5 MeV *α* particles fall more rapidly than the rest of the distributions (Fig 8c). Moreover, BNC sample distributions present greater dispersions compared to accelerated particles. Roundness (Fig 8d) in irradiated Lexan foils shows values close to 1, and the same occurs with Si doped foils, with >90% of quantified objects presenting values of 1. On the other hand only 80% of objects from BNC samples exhibit values close to 1. Considering Area, Aspect and Roundness, *α* tracks would appear to be morphologically similar to the ones generated by the BNC reaction. Moreover, we conclude that the significant dispersion among images in the selected parameters corresponds to the variety of incident particles, energies and emission angles.

Heterogeneity (Fig 8e) distributions show that tracks generated by particle beams are less uniform in pixel-intensity since detected objects present values close to 1. This intrinsic inhomogeneity disappears in Clumpiness (Fig 8f), so the objects have greater contrast gradient between proper pit and background. Both in Heterogeneity and Clumpiness distributions it can be observed that the tracks corresponding to monoenergetic beams are more similar to each other compared to those coming from BNC events.

### Influence of light and focus settings

**Fig 9:**
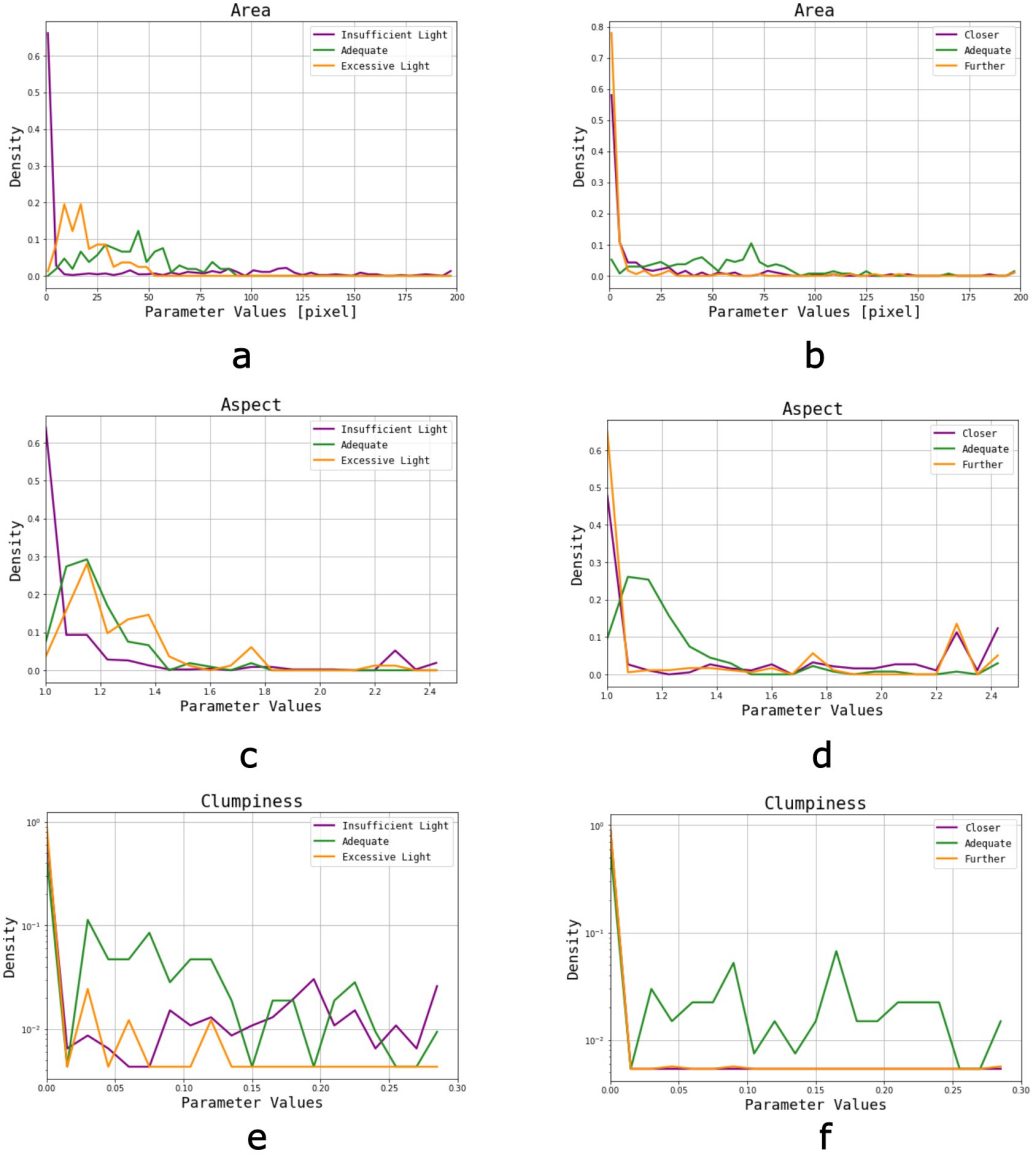
Parameter distributions of images acquired from the same area, but changing the light intensity or focus among them. a-c-e) Area, Aspect and Clumpiness distribution for light scanning, b-d-f) Area, Aspect and Clumpiness distribution for focus scanning.

For poorly illuminated images there is a significant increase of small Area objects due to background pixels (usually white) that lower their grey intensity appearing as irregular shadows, and get quantified. When analysing overly illuminated images, a shift towards smaller Area objects is observed (Fig 9a), since tracks are artificially shrunken. With an inadequate focus the results are similar (Fig 9b), especially for a long sample-lens distance, which causes a lack of border definition resulting in foreground isolated pixels, as well as dark spots or shadows. Observations in the distribution of Diameter values are consistent with Area distributions.

Since isolated pixels and small clusters have Aspect Ratios of 1, the lack of light and a wrong focus produce an accumulation of objects with Aspect<1.05 (Fig 9c and 9d). These smaller objects tend to disappear with an excess of light, so larger objects with less circular edges gain importance in the corresponding histogram. Moreover, roundness distributions are consistent with Aspect values. Artifacts and track distortion resulting from a wrong focus impact as higher Aspect and Roundness values, indicating that these quantified pits have less circular shapes.

Clumpiness (Fig 9e and 9f) values for both extreme light and focus conditions accumulate mostly in the first bin (<0.008). A saturation of light causes smaller and brighter pixels to disappear, resulting in a greater amount of objects with homogeneous pixel intensity. On the other hand, insufficient illumination results in an excess of isolated pixels and small clusters with even intensity values along the object. After the erosion filter, small objects will not be detectable, and so the first bin in Clumpiness becomes more relevant with respect to normal conditions, especially for excessive amounts of light. Since lack of light generates artifacts with intensity inhomogeneities in the entire object (not mainly around the edges, as regular tracks), there is an increase in higher Clumpiness values. Furthermore, tracks observed in images acquired with a wrong focus appear more homogeneous (similar and lighter pixel intensities throughout the objects), and the change in object intensity due to a wrong focus, in addition to a higher amount of isolated pixels in object edges, could explain this behaviour. Heterogeneity distributions are consistent with the observations in Clumpiness. This parameter appears to be more sensitive to images acquired with insufficient light, where an increase in quantified isolated pixels is evident.

In summary, distributions proved to be sensitive for energy and light intensity in images resulting from monoenergetic particle irradiations, especially those from size-related parameters. Nonetheless, this sensitivity is not as relevant in BNC distributions. As mentioned before, particles with multiple energies arrive at the NTD due to the distance between the BNC reaction site and the surface, as well as the emission angle. The fact that BNC distributions are wider allows the technique to be somewhat robust towards eventual light variations in acquisition conditions. Additionally, these distributions were a promising criterion to continue with the task of image classification without pursuing more time-consuming algorithms.

### Classification algorithms

With the SVM’s confusion matrix (Table 3), the metrics of interest were calculated. Accuracy was measured as the ratio between correctly classified images (true negatives and true positives) and the total amount of images, revealing a value of 93%. The second metric of interest, precision, was calculated as the ratio between images correctly classified as Accepted (true positives) and the total of images classified as such. This metric resulted in 93%, favourable for quantifying mainly adequate images.

**Table 3:** SVM’s confusion matrix.

		Prediction	
		Rejected	Accepted
Label	Rejected	7687	595
	Accepted	544	7745

The numbers in each box represent the amount of images predicted using cross validation as one class, compared to their actual ground truth.

**Fig 10:**
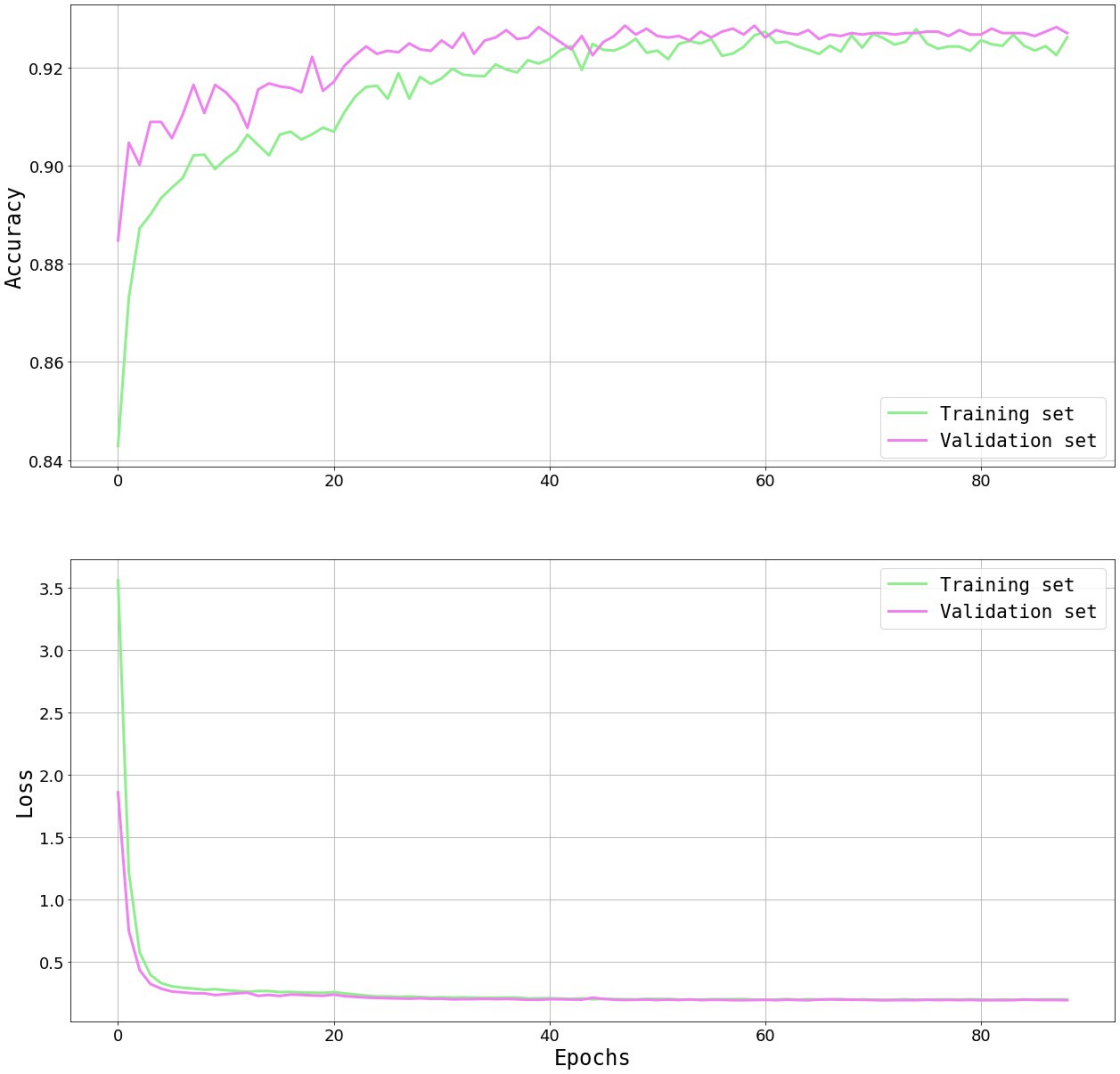
Progression the NN’s training process, comparing results in training set and validation set. a) Accuracy, b) Loss.

The NN was trained several times (≈80 epochs each time, using Google Colab) adjusting the number of neurons, layers, optimization algorithms, learning rate, among others. Learning curve (Fig 10a) and cost function (Fig 10b) were plotted in order to control the training process, displaying an acceptable performance. By comparing the evolution in validation and training set an assessment can be made regarding the capability of generalization. We chose the architecture where the validation performance curve follows a similar trajectory as the training curve, ensuring better generalization, and where the validation accuracy is larger, ensuring better performance in unseen data.

NN confusion matrix (Table 4) was generated using the validation set, obtaining an accuracy of 94% and a precision 95%.

**Table 4:** NN’s confusion matrix.

		Prediction	
		Rejected	Accepted
Label	Rejected	1579	81
	Accepted	116	1539

The numbers in each box represent the amount of images corresponding to the validation set predicted as one class, compared to their actual ground truth.

Given the seemingly similar performance between both models, a further comparison was performed. The first analysis was a ROC curve, which plots the true positive rate (TPR, truly acceptable images predicted as such, over the totality of images labelled as Accepted) against false positive rate (FPR, images wrongly predicted as acceptable, over the totality of images labelled as Rejected) for different threshold values. The threshold works as the decision boundary given the model’s output: values>threshold will be classified as positive (Accepted), whereas values<threshold will be classified as negative (Rejected). For an ideal model (with an ideal dataset), there will be a threshold value at which classes get perfectly separated (TPR = 1, FPR = 0), but at higher values the amount of false positives increases while the true positives remain the same. Conversely, at lower values the amount of true positives decreases, while no rejected image gets classified as accepted. To simplify the comparison, the Area Under the Curve (AUC) is commonly employed to characterise the analysis, where the goal is to obtain an AUC closer to 1 (with AUC = 1 being the ideal model described above). In reality, perfect models are not achievable and there is always a compromise of TPR and FPR, so the expectation is to obtain values>0.5, seen as better than a random classification.

**Fig 11:**
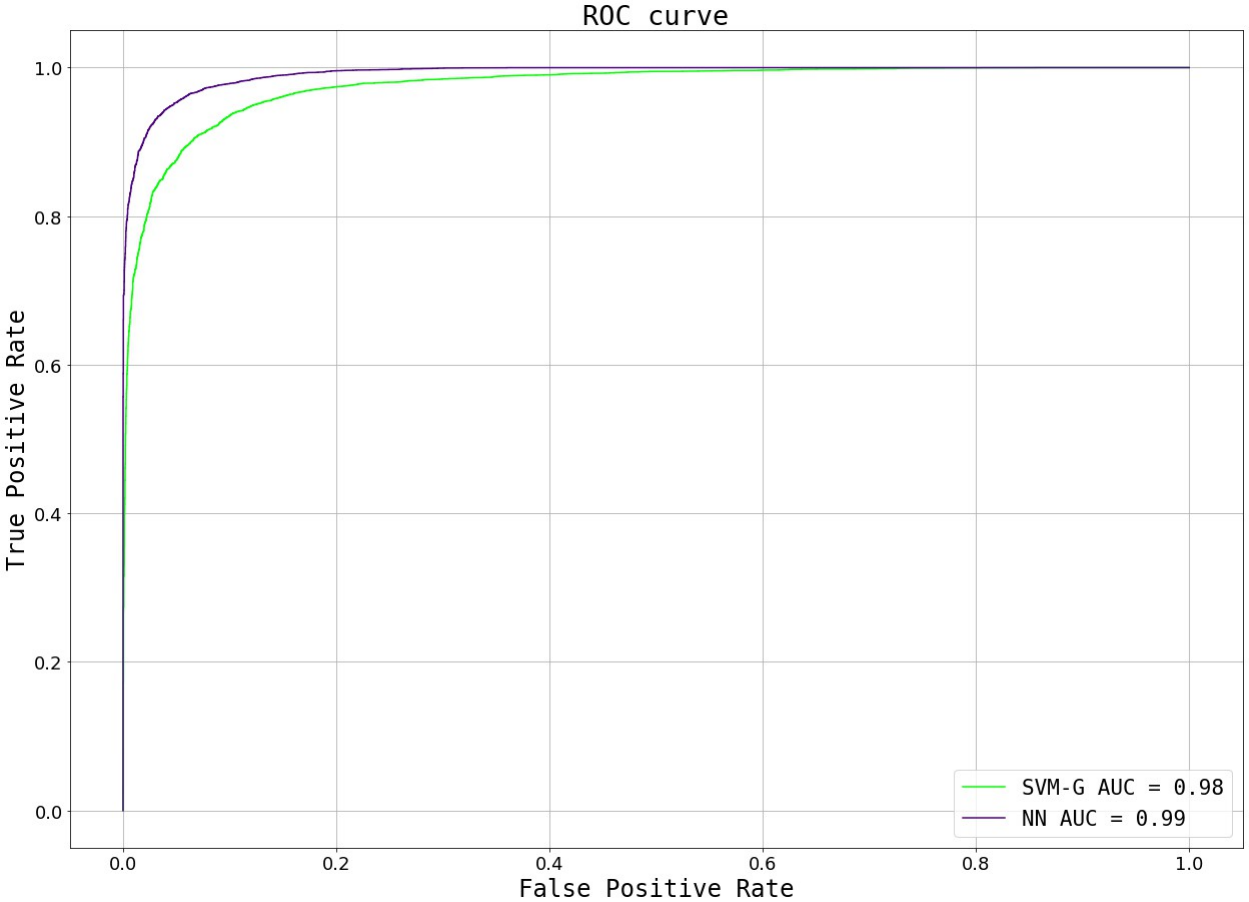
ROC curves for both the SVM and NN trained models, with their corresponding AUC values.

**Fig 12:**
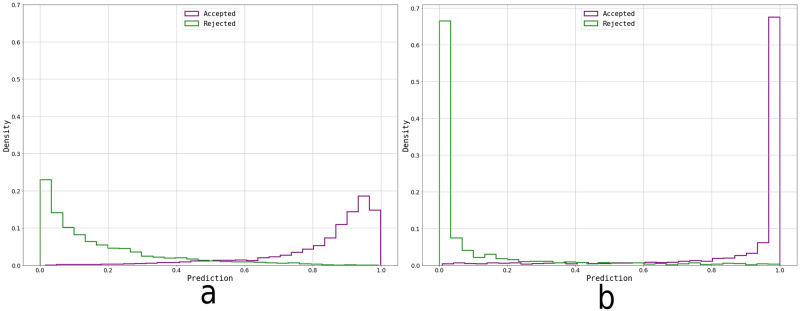
Prediction probability graphs based on the ground truth, for both selected models. a) Prediction histogram resulting from the cross-validation output (16571 data points) with the SVM model, b) Prediction histogram resulting from the validation set’s (3315 data points) output with the trained NN.

When comparing SVM vs NN’s ROC curves (Fig 11), particularly their AUCs of 0.98 vs 0.99 respectively, it is appreciated that NN’s performance is slightly, but not significantly, better at detecting more acceptable examples as such without misidentifying inadequate images. This metric alone does not provide an absolute result on which model to use but, together with the Prediction histograms (Fig 12), encourage us to select the NN method as our classification algorithm.

The Prediction histograms show the output of the model for each image. A sigmoid was applied to the SVM’s decision function to obtain a prediction between 0 and 1 (Fig 12a). It can be observed how the NN is more confident in its predictions by correctly clustering the images to the edges (Fig 12b) while the SVM centers more images around the decision boundary (Fig 12a) reflecting a higher degree of uncertainty about the true class for certain types of images. From this, we can conclude that the NN performs a better job at separating the two types of images using the 31 principal components as features.

**Fig 13:**
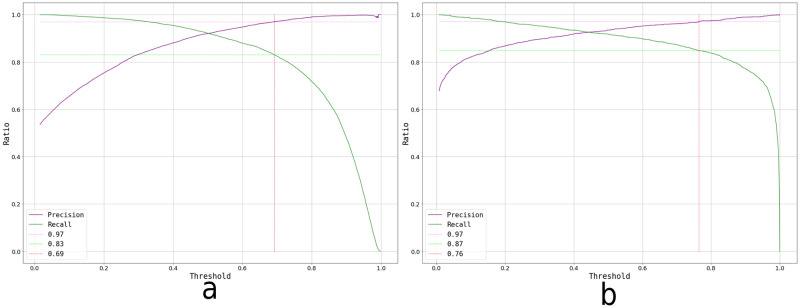
Precision-recall curves. a) SVM, b) NN.

Additionally, precision-recall curves were plotted for both models, in order to evaluate the possibility of changing the decision threshold to increase the precision up to 97% (Fig 13). The decision of improving precision up to this value was based on a subjective evaluation of these curves: the goal was to keep the highest amount of useful images without discarding too many adequate ones due to a wrong classification. Recall is a performance metric complementary to precision, where the true positives are compared to the totality of positive labels. By raising the threshold, an increase in false negatives occurred, resulting in a lower recall. Complementary, decreasing the threshold results in a higher amount of false positives, hence a reduction in precision. Given the goal of this work, the optimal algorithm ought to have a high precision, and the best achievable recall. Changing the threshold in the NN resulted in a higher recall (87%), compared to the same modification in the SVM (83%). As a result of these two comparisons, the selected algorithm was the NN with a threshold of 0.76. Typically, during an autoradiographic analysis, around 50 images are acquired for each region of interest. Assuming 45 adequate (true positives [TP] + false negatives [FN]) and 5 inadequate (true negatives [TN] + false positives [FP]) acquired images, these precision-recall values result in approximately TP = 39, FN = 6, FP = 1 and TN = 4. Thus, without missing too many adequate images (6), only one inadequate image would be considered in the analysis, which statistically has no significant impact on the final boron concentration value.

**Fig 14:**
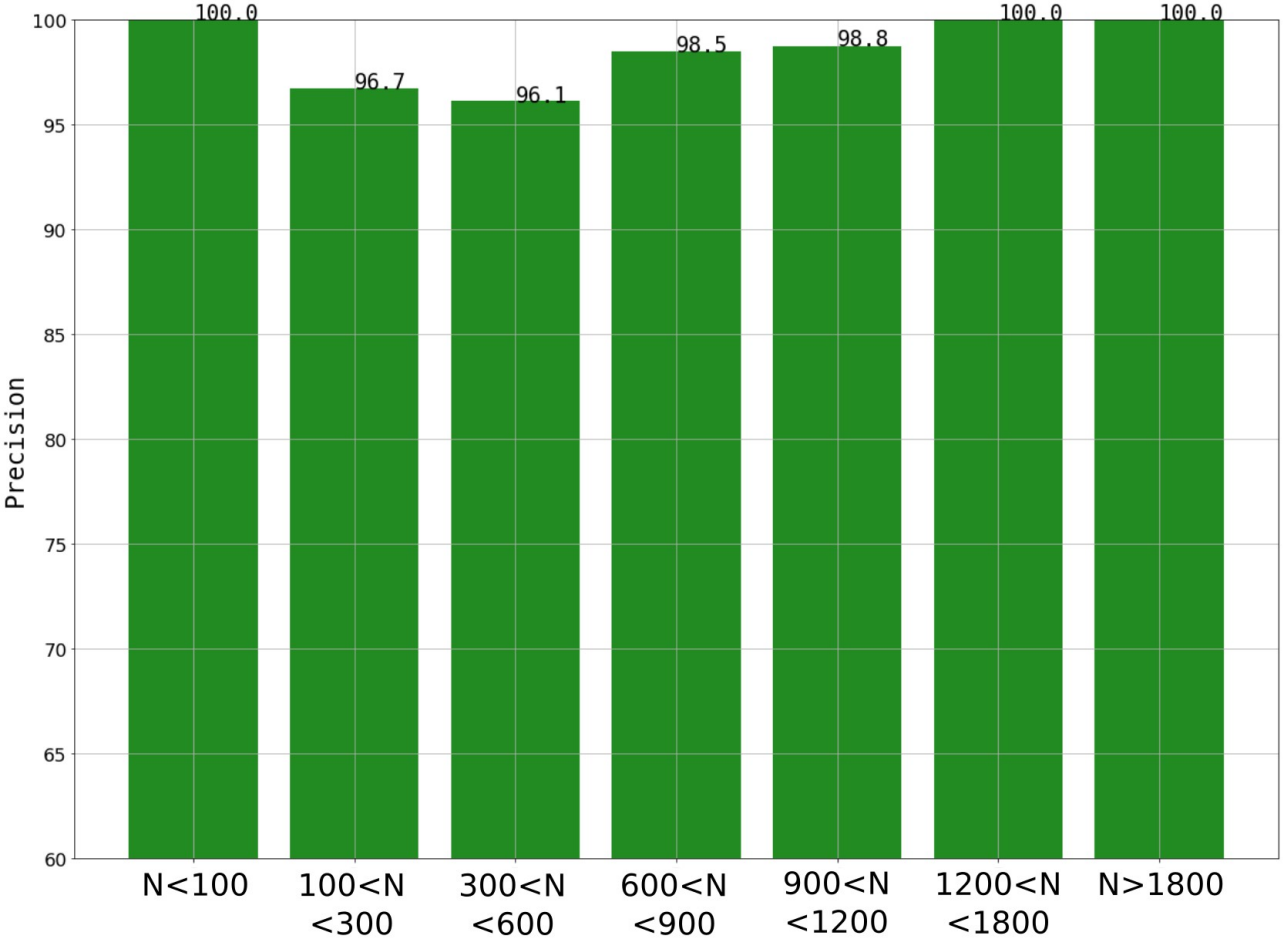
Precision vs amount of tracks (N) in each validation set’s sample.

To further analyse the model’s classification performance, samples in the validation set were grouped in terms of their track count. Measuring precision in these subsets (Fig 14) revealed that the algorithm is fairly adequate for classifying images independently of their track quantification. This result is of great relevance, since boron concentration in biological samples (i.e., track amount in the autoradiographic images) could vary considerably depending on the boron compound and the histological characteristic of the tissue. Eventually, different algorithms could be trained for varying amounts of tracks.

The confusion matrix of the test set (Table 5) revealed metrics of 91% for accuracy and 96% for precision, comparable to those obtained using the validation set and similar to a human classification performance. These final performance metrics make for a promising algorithm to incorporate into the laboratory’s workflow, thanks to the high levels of both precision and recall, which were aligned with the motivation of this work.

**Table 5:** Test set’s confusion matrix.

		Prediction	
		Rejected	Accepted
Label	Rejected	2075	65
	Accepted	315	1688

The numbers in each box represent the amount of images corresponding to the test set predicted as one class, compared to their actual ground truth.

**Fig 15:**
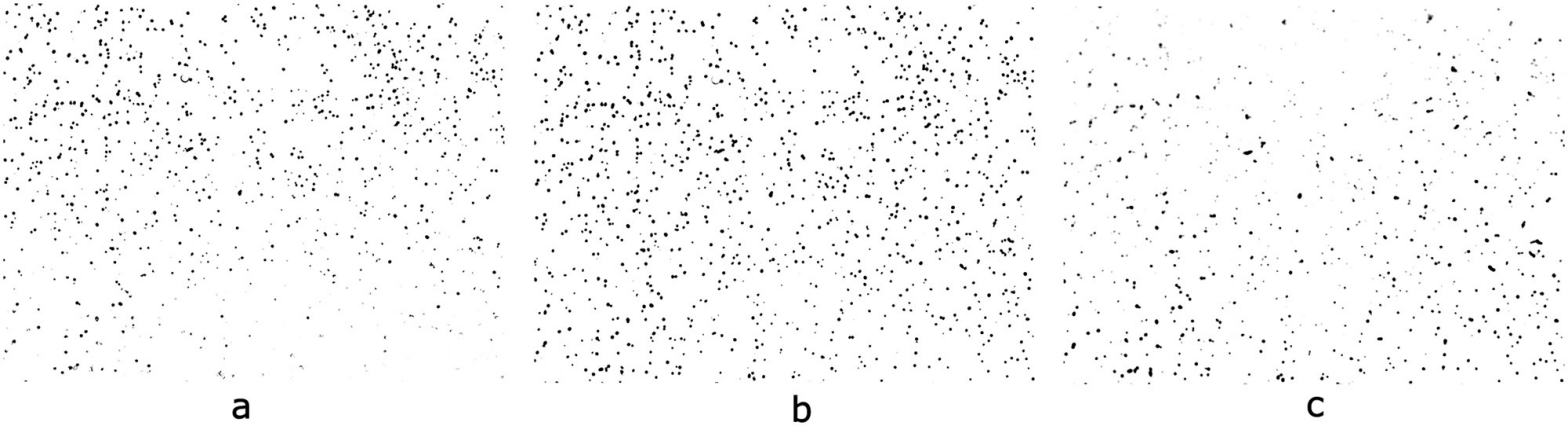
Acceptance probability (P) for each image acquired with a different lens-sample distance, and the corresponding amount of quantified tracks (N). a) acquired under the wrong conditions: P = 0.0867, N = 1094, b) acquired under correct conditions: P = 0.9994, N = 1192 (c) acquired under the wrong conditions: P = 0.0135, N = 1001.

Furthermore, an extra test was performed using 3 new images of the same area, but varying the lexan-lens distance (Fig 15). This serves as an application example where using an inadequate image would lead to a wrong track quantification.

As it is, the algorithm works for the experimental conditions set for this laboratory, but could be extended for different neutron fluences given the tracks are still able to be segmented. The same can not be said for a different etching condition (time, temperature or composition), or a change in the microscope, if they imply a difference in the morphology or the visualisation of the tracks.

The addition of this automatic verification step is useful both for relieving part of the image visualisation work, and for training new users in the process of learning how to acquire adequate images. Due to the sensitivity of some parameters to different acquisition conditions, it should be possible to extend this binary classification to a multiclass one to allow the distinction among potential problems (i.e. let the user know whether the image should be acquired with a different light setting, or to adjust the focus), but images would have to be re-classified and the dataset would most likely have to be extended to balance these potential classes.

The use of machine learning algorithms is rising among different applications due to their ability to process large amount of data, extract new information, and optimize the analysis. Because of the interdisciplinary nature of BNCT, there are numerous applications to explore. In particular, a neural network-based dose prediction method has been recently reported [36]. As for our laboratory, further work will be devoted to the use of Convolutional Neural Networks, widely used for medical applications [37], for tasks such as the segmentation of nuclear tracks and cell compartments.

## Conclusion

Notwithstanding the fact that etched pits in polycarbonate seemed to be fairly regular, a great amount of information was obtained by characterising each detected track with a series of morphological and gray-scale uniformity parameters. The distribution of such parameters provided relevant information regarding the physics behind the nuclear tracks generation and the image acquisition conditions. This allowed not only for the automated verification of autoradiographic images, but also for the reduction of the input information and the time consumed on training machine learning algorithms.

A simple but adequate image classification algorithm was developed, by performing a manual feature extraction step, in which track-parameters distributions were used to represent each image. These parameters proved to be useful for identifying wrongly acquired images, and the algorithm was able to classify new images with a performance comparable to that of a human expert.

We have recognized the potential of including machine learning methods in our workflow. Further work will be devoted to extending their use to different steps in the autoradiographic analysis, such as the segmentation of tissular structures, or quantification of nuclear tracks in images with cellular or tissular contours.

## Supporting information

S1 Dataset Parameter dataset. Complete dataset of the original 22349 images represented by the aforementioned 36 parameters (mean, median, interquartile distance, standard deviation 5th percentile and 90th percentile of the track area, diameter, aspect ratio, roundness, heterogeneity and clumpiness distributions). This dataset also includes information of each image’s track density, corresponding label and name.
(CSV)
